# On the Calomel Treatment in Algide or Asiatic Cholera

**Published:** 1849-09

**Authors:** 


### ECLECTIC DEPARTMENT,

### AND SPIRIT OF THE MEDICAL PERIODICAL PRESS.

*On the Calomel Treatment in Algide or Asiatic Cholera.* By ARCHIBALD HALL, M. D., L. R. C. S. E., Lecturer on Materia Medica, M'Gill College.

An apology for devoting, at this time, a considerable portion of our eclectic department to articles relating to epidemic cholera, will not, we presume, be required by many of our readers. Aside from the intrinsic interest of the subject in a scientific point of view, the fact that this fearful disease is still prevalent in many parts of our country, and the high probability that it may continue to prevail in different sections for some time to come, render it desirable to extend, in so far as practicable, all important practical information respecting it, and especially to diffuse the results of the practice of different practitioners, as respects its treatment. In selecting articles relating to this disease, our rule has been, and is, to choose, 1st, those which commend themselves from the internal evidence they afford of their value; and, 2d, those emanating from persons well known by us individually, or by the profession at large. The author of the following paper is Editor of the British American Journal, and our personal acquaintance, as well as his high character as an honorable and distinguished member of the profession, led us to peruse it with not a little interest. The results of his experience certainly go strongly to sustain the mercurial plan of treating the disease—a plan upon which the majority of practitioners who have been practically conversant with the disease, appear to be united.—*Editor Buffalo Medical Journal.*

Dr. Graves, of Dublin, in his recent second edition of his "clinical lectures," alluding to the treatment of Asiatic or Algide Cholera, by calomel, thus remarks: "Before we proceed further, I may observe, that the principle on which the calomel treatment was employed in cholera arose from almost constantly observing that there was a total deficiency of bile in the stools. Soon after the supervention of an attack, the alvine discharges were observed to be white, and without the slightest tinge of bile, and on this very remarkable symptom practitioners dwell almost exclusively, thinking that the patient's only chance lay in restoring the action of the liver. Now it is obvious that the absence of bile in the stools is no more a cause of the disease than is the deficiency of urea in the kidneys, or of serum in the blood. Viewing the disease in this light, it would be just as reasonable to give a diuretic to restore the secretion of the kidneys, as to give calomel to produce a flow of bile, &c., &c. I have, therefore, no hesitation in saying that the calomel treatment has no claim to merit on the ground of theory; and, as far as I have observed the results of it in this country, it seems to be of no practical value in the treatment of cholera."

With every deference for the high character of Dr. Graves, and to that position in the profession to which his talents justly entitle him, and which he has fairly earned, the experience of the calomel treatment in this city, during the present epidemic, is so completely at variance with the above recorded opinion, as to lead to a doubt whether it was fairly pursued in Ireland, or, that Dr. Graves has (what I do not believe) underrated it as a means of relief, for the purpose of advancing a new method of treatment, which might be deemed peculiarly his own. I allude to the treatment by *acetate of lead and opium* proposed by that gentleman.

Into the remote cause, inductive of that peculiar condition of the system characterised by the train of symptoms known under the name of Asiatic Cholera, it is not my present purpose to inquire. It is a matter of little moment whether it be of an endemic or epidemic origin; whether terrestrial or atmospheric, whether depending on disturbed electrical equilibrium or of a fungoid character; at the present moment I deal with its effects, and no one who has witnessed the disease can question this statement, that superadded to the ordinary phenomena, there is witnessed a manifest impression upon the nervous centre of a depressing nature, and that this impression is antecedent to the evolution of the several symptoms which follow, and would appear to be inductive of them, varying in intensity, however, in different cases; prostrating the vital and dynamic forces at once in some, effecting its purpose more slowly but not less surely in others, and in a third class manifesting itself in a more manageable foim in the shape of the diarrhœa now so prevalent.

Examining again into the pathological changes induced by the disease, in by far the majority of cases, we find, with the exception of congestion of the internal blood vessels, but insufficient causes of death. Sometimes the mucous membrane of the alimentary canal is found to be inflamed, at other times not; sometimes pulpy and thickened, at other times blanched and anæmic. Dr. Boehm, at present so worthily supplying Dr. Deiffenbach's place at Berlin, in a recent work shows that "the chief pathological alteration of the mucous membrane in cholera consists in a desquamation of the epithelium,' by which it is often altogether thrown off, and that the process commences at the lower portion of the ileum, where the injection of the blood vessels is most distinctly seen. The liver is occasionally found congested, at other times perfectly healthy in appearance. The kidneys have always been found healthy, although suppression of urine is one of the most marked symptoms of the disease. The gall bladder is almost always found distended with bile. The bladder contracted; and the brain and spinal cord most usually normal in their appearances. What, then, is the cause of death, for there is nothing in these pathological alterations, which should not in analogous cases, afford the fairest anticipations of successful treatment? Take we into consideration the sudden collapse, the enormous drain upon the circulating fluid, the complete arrest of secretion, whether in the salivary glands, pancreas, liver or kidneys, and the utter prostration; couple all this with the deficient and inadequate pathological changes, and we *must* look to an impression on the nervous centre as the *first cause*, the active agent, inductive of the mischief, and all the symptoms concur in pointing out the ganglionic system, or the great sympathetic, as the one immediately affected, influenced in its innervation, and destroying, by consequence, the equilibrium of the circulation, and the tone of the capillaries, especially in the intestinal canal; and impeding secretion, and, to a marked extent, absorption.

Based upon the observations made, would appear to arise three important indications:—1st, The restoration of the ganglionic system to its pristine condition; 2d, The arrest of the vomiting and purging; and 3d, The re-establishment of the various secretions by excitation of the glandular viscera. Experience has amply illustrated this fact, that the attainment of the first of these indications cannot be effected by stimuli directly applied. No line of treatment in cholera has proved more signally unsuccessful than the stimulant one unaided. Ether, ammonia, alcohol in its various forms, camphor, opium in small and frequently repeated doses, capsicum, &c., have all failed. This is too well established to admit of dispute. The first indication, then, must be sought to be fulfilled by the exhibition of medicines calculated to secure the second and third, but especially the latter, although the exhibition of stimulants is at the same time necessary and proper to sustain the powers of the system, and to *secure time*. Will what is called the saline treatment effect this? Assuredly it will not; and it is difficult to conceive the precise object to be attained by its adoption. It was at one time supposed that the saline constituents of the blood became diminished in their natural quantity. The experiments of Drs. M'Lagan,

Christison and Robertson, of Edinburgh, prove the reverse of this. What is called the Russian mode of treatment subserves no better end; and the repeated exhibition of opium in the stage of collapse, appears to me to be most unlikely to answer any good purpose. There is no medicine with which we are acquainted more surely adapted to suspend secretion than opium, and there is therefore none more unfitted for protracted employment in cholera, in which secretion is suspended,—and, on the contrary, no medicine seems better adapted to fulfil the several indications than mercury, effecting its purpose by the erethism consequent upon its steady administration, in which the whole system participates.

Calomel in large doses is well known to be a powerful sedative in cases of irritability of the stomach, attended with nausea and vomiting; and in the treatment of sporadic and even infantile cholera, no medicine which we possess presents higher claims to consideration. The experience of the first physicians in this Province and the United States might be cited in its favor; and that it should present itself prominently to consideration in the management of an analogous condition of the stomach in the algide variety of the disease, is by no means surprising. Having been exhibited in the commencement of treatment, when symptoms demand it, in the manner and for the purpose indicated, it should become an object of equal importance to secure its constitutional influence by its steady administration in smaller, frequently repeated doses, combining it with stimulants, especially in the cyanic stage, with the twofold view of exciting the absorbents, and securing time by sustaining the vital powers. When exhibited in the large doses, at the commencement of treatment, especially if combined with morphia, it will be found to act in the majority of cases in the most satisfactory manner, allaying the vomiting almost instantaneously; while we afterwards seek to establish its constitutional effects by its exhibition in smaller doses, repeated every half-hour or hour, in accordance with the necessity of the case, combined with camphor or assisted by sulphuric ether, or some other stimulant of a similar character.

In my own practice, since the epidemic which now prevails commenced, I have had the good fortune to encounter, up to the present period, but ten cases of Cholera of the Asiatic variety; and as I have treated them on the principles laid down, I will let the results speak for themselves. To avoid prolixity, I will be as brief as possible in the descriptions, which will be given chiefly for the purpose of pointing out the stage of the disease at which the treatment commenced.

Case 1.—July 3, 6 P. M.—I was requested by Dr. H. to visit his wife, who was suffering under severe diarrhœa, which had existed during the greater part of the day. The evacuations were frequent, occurring about every half hour, and of a bilious chatacter, attended with griping and some nausea; her pulse was regular but weak, and her countenance was natural. I requested Dr. H. to give her some powders containing pulv. cretæ. comp. c. opio., to which I felt desirous of adding calomel, but to which objection was made, in consequence of the debilitating effects which she supposed it induced, and which she remembered having experienced at an early period of her youth. Satisfied, *pro tempore*, with the prescription, I left, and visited her at 10 P. M. I now found her in a state of incipient collapse, eyes sunk, nails blue; arms, legs, and feet cold, and the legs cramped; and immediately before my entrance, she had had a copious evacuation, presenting the rice water character, and she had vomited several times. The following prescription was immediately ordered:—

℞. Hydrarg. Chloridi gr. v.
Pulv. Gum Camph. gr. ij. Fiat pulv.

One to be taken every fifteen minutes until the vomiting ceased.

11 P. M.—Mrs. H. has had four evacuations, of the rice water character, since last visit, but much diminished in quantity; the irritability of the stomach still continues. The cramps have subsided, and the pulse is rather weaker; ordered ice in the mouth; and the powders to be continued every hour, with small quantities of brandy and water from time to time.

12 P. M.—Vomiting and purging quite ceased; the former from the moment of using the ice; the powders still to be continued.

July 4, 3 o'clock, A. M.—Considerably improved in every respect; has had one motion, of a bilious character, and moderate in quantity. The medicines were now discontinued, and calf's foot jelly, with beef tea, substituted, each in small quantities, from time to time. From this period she convalesced from the attack, although a fever, of a remittent type and mild form, supervened in the course of a couple of days, and in the course of the week subsided.

The above case was a mild one, and yielded readily to the treatment employed. It was one of the earliest cases in this city.

Case 2.—July 8.—Was this morning requested by Dr. Scott to visit Mr. T. S., in consultation. This gentleman had been suffering under diarrhœa, for at least the day preceding the attack, and had partaken on the day previously of a salmon dinner. The diarrhœa was increased by this, and continued during the night, accompanied by vomiting. Dr. Scott was summoned to his bedside about 5 A. M., and prescribed two or three large doses of calomel. When seen at 9 A. M., the vomiting and rice water purging still continued: he was cold, covered with clammy perspiration; countenance collapsed, tongue cold, skin blue, and the skin of the fingers in longitudinal wrinkles; pulse almost imperceptible at the wrist; cramps in the legs; suppression of urine, tinnitus aurium, and whispering voice.

The calomel and camphor were now advised, in doses of 5 grains and 2 grains respectively. They were exhibited in pill form every hour, conjoined with sulphuric ether in camphorated mixture, and brandy and water; external applications were also assiduously applied.

9 P. M.—Found the patient but little improved; the vomiting and cramps had ceased, but the rice water ejections still continued. Pulse almost imperceptible. In every other respect, there was no change. The administration of the medicine in pill form was now discontinued, and that of powder substituted, given every half hour; steadily pushing on the collateral treatment.

July 9, 9 A. M.—Patient improved in every respect; heat of surface returned; tinnitus aurium gone; voice restored; pulse full and soft; countenance improved, and blueness disappeared; reaction had set in during the night, and he had made water once, and had had two evacuations, of a dark bilious character. He was an altered man in every respect, and expressed himself to that effect. A mildly nutritious diet was now ordered, with other necessary directions. After we left, I am informed, he got up, shaved, and dressed himself, and committed other imprudencies. I was sent for on the evening of the 10th, by Dr. Scott, and found him dying.

He was in a semi-comatose state, and was sinking under a typhoid attack, which, I have not the slightest doubt, was due as much to his own imprudence as to an enfeebled constitution.

In this case the recovery from the state of collapse, and this, too, of the worst description, was complete, and can be attributed only to the steady employment of the calomel.

Case 3.—Mrs. R. P. had been suffering under a neglected diarrhœa for several days. Shortly after an attack of vomiting, I was summoned to see her, on the 14th July. She had several "*watery*" stools, as she called them, and now, in consequence of the vomiting, sought relief. Symptoms of collapse were setting in, and there had been slight cramps in the legs, and considerable griping. One dose of ʒss of calomel, with gr. i. of muriate of morphia, sufficed to allay the vomiting, in the course of half an hour; the calomel was repeated at the expiration of that time, and was followed by calomel and camphor, in three grain doses of each, every three hours, under which, the symptoms completely yielded. This patient became salivated, a matter of little moment, when the question involved is one of life or death.

Case 4.—Mrs. M'C had been seen by Dr. Nelson in my absence on the evening of July 16; on the morning of the 17th I was called to attend her at 7 A. M. She had been passing rice-water stools during the night, and the stage of collapse was fairly setting in, as indicated by her countenance, her pulse, and her skin. The same treatment was pursued as in the preceding cases, and the result was of the same character, as far as the disease itself was concerned, for which I was consulted. She has perfectly recovered.

Case 5.—I was called at half-past four A. M. to visit Mrs. A. She was laboring under vomiting, purging, and cramps of the legs. The diarrhœa, however, had been severe for the preceding six or seven hours, and gradually assumed the rice-water character, and the appearance of the countenance, skin, and pulse, indicated the supervention of speedy collapse. A similar line of treatment was adopted as in the last cases; and although the diarrhœa was more obstinate than usual, yet after the gums were slightly touched it yielded, and she is now perfectly recovered.

Case 6.—July 25.—At 8 P. M. I was hurriedly called to see Mrs. McK., aged 70. When seen she was laboring under all the symptoms of collapse with vomiting, and rice-water stools, superadded to which were violent cramps of the lower extremities. I left with her three powders; No. 1 containing ʒss of calomel and gr. 1 of mur. morph.; No 2 ℈j. of calomel, and No. 3 grs. x of calomel. On my second visit, one honr after, the vomiting and cramps had disappeared; and I prescribed calomel combined with camphor, every hour. Before this time she had passed several rice-water stools; but on my visit next morning I found that the diarrhœa of peculiar character had subsided, and she had passed a couple of dark colored stools. She had attained the age of 70 years, and died in the course of twenty-four hours after, of exhaustion. In this case recovery from the state of collapse was also complete.

Case 7—July 25.—At 9½ A. M. I was requested by Mr. M. to visit a young man in his employ, named W. A. He had been suffering under a severe diarrhœa for the five preceding days, for which he had obstinately refused to take advice. Finding himself worse than usual, and vomiting coming on, he consulted Dr. McCulloch about 6 A. M., who prescribed for him, with instructions to let him know his state at 9 A. M., if not better. In the absence of Dr. McC., I visited him. Collapse was just supervening; his nails were blue; eyes sunk; pulse small but distinct; vomiting; rice-water stools, and severe cramps of the lower extremities. I gave him immediately ʒss. of calomel, and 1 gr. of morphia, to be followed by ℈j. of calomel in a half hour, and 10 gr. more in another half hour; and left him several powders, each containing 3 gr. of calomel and 3 of camphor, one to be given every hour. Brandy and water was also ordered, with the usual external applications, sinapisms, &c. At 2 P. M. the vomiting had ceased, and the diarrhœa had moderated, although the character of the stools continued the same. The pulse was more feeble; I continued the powders every half hour. At 6 P. M. I considered the young man to be dying. He was cold, pulseless, in a state of semi-insensibility, and unable to articulate or to swallow, with his eyes fixed, and conjunctivæ injected. In consequence of the supervention of these fatal signs, the attendants had intermitted the medicines for the preceding hour or two. He was now seen by two friends, who, considering that while there was life there was hope, determined to persevere with the treatment pursued during the day, and at the same time continued the external means. In the course of about three hours, he passed a couple of bilious, dark stools; and about 10 o'clock, they felt pulse at his wrist. Re-action had commenced. Dr. M'Cullough was found in the neighborhood, and requested to visit him; treatment appropriate to this stage was now adopted, and the young man has recovered from the cholera, although he still labors under consecutive typhus fever.

Case 8.—July 26.—I was called at 11 A. M. to attend G. M. He had attained the age of 40 years, but possessed the constitution of a man of 60, from previous hard living. He was a storeman, and although laboring under a disease of no ordinary character, yet attended to his duties, till compelled by his condition to give up. I saw him about 11 o'clock, about seven hours after the commencement of the severe diarrhœa alluded to. He was walking about, had tinnitus aurium, whispering voice, a collapsed countenance and feeble pulse. I immediately ordered him to his bed, and left him ʒss. of calomel with morphia, the calomel to be repeated in half an hour; immediately after I left I understood he began to vomit, and was severely attacked by cramps. On my return in the course of an hour and a half, I found that the vomiting had ceased after the exhibition of the calomel; the cramps continued severely, with the purging, which was of the rice-water character, or as his wife termed it, like "sour ginger beer." Three doses of calomel and camphor were now ordered, one to be given every hour. At half-past 2 P. M. I again visited him, and found but little alteration in the symptoms. The blueness of the skin was complete, pulse more feeble, and the state of collapse more perfect. The calomel and camphor powders were now ordered to be administered every half hour, alternating them with sulphuric ether in camphorated mixture; port wine was also given along with the powders, and ice in the mouth to allay thirst. At 5 P. M. the diarrhœa had subsided to a marked extent, and the vomiting was wholly allayed; he continued gradually sinking until about 2 A. M. next morning, when he died.

This case is the only one which has occurred to me, in which the recovery from the state of collapse did not take place.

Case 9—July 29.—At 4½ A. M. I was called out of bed to visit the child of Mr. G. M., residing in one of the most infected parts of the Quebec suburbs. The child, 2 years of age, had been seized with vomiting and severe purging during the night, which excited alarm when the latter was found to assume the rice-water character. Dr. Deschambault, living in the neighborhood, was forthwith called in, who prescribed for the child; I was afterwards sent for at the hour specified, the parents having been for many years patients of my own. The child was quite cold, with a filiform pulse, and collapsed countenance. Shortly before my arrival, it had a stool, which I saw, of decided rice-water character. The vomiting was still urgent. I ordered the child four grains of calomel, to be given every half hour until the vomiting ceased, leaving four such powders, and afterwards the calomel in two grain doses, to be exhibited every hour, alternated with a tea-spoonful of a mixture containing equal parts of camphorated mixture and water. When seen at 10 o'clock, Mrs. M. had only found it necessary to administer three of the powders, but the camphorated mixture had been given regularly. The child had improved in every respect, and mild nutritious diet was substiuted for the medicines.

Case 10—July 29.—G. W. had luxuriated on the vegetables now so common and so cheap, and despised the restrictions of the Profession in regard to them. After a hearty dinner, of which green peas and cucumbers formed a large portion, assisted by a tumbler or two of beer, he found that the diarrhœa, which had given him no uneasiness fon the preceding two or three days, became suddenly aggravated, with considerable griping and nausea. He tried to allay the symptoms by burnt brandy. About 7 P. M. vomiting commenced, and the stools became more watery; and alarmed by a cramped feeling in his legs, he sent for me, and I saw him about 8 1-2 o'clock. The symptoms had rapidly progressed since he sent off his messenger. The skin of his arms, legs, and face, was blue, and covered with a cold, clammy perspiration; voice whispering, tinnitus aurium, severe cramps in the lower extremities, and occasionally in the arms and fingers, gripings in the bowels, which were opened every 15 minutes, the stools presenting a rice-water character; tongue cold to the touch; incessant retching, but bringing up nothing. Pulse small and thready, countenance sunk, and the whole appearance indicative of decided collapse. The same line of treatment was pursued as in the other cases; the irritability of the stomach quickly subsided. In the course of about six or seven hours, he passed a bilious evacuation, the pulse rose, and reaction set in. This case also recovered.

In the details of the cases, I have sought as much as possible conciseness; my intention having been rather to illustrate the treatment adopted, and to point out the stage of the disease at which it commenced, than to give a long history of symptoms, which in the present disease do not vary essentially in the different cases. I have only incidentally alluded to the collateral external means employed; these consisted in the free use of sinapisms to the spine and epigastrium, and hot applications of various kinds to the extremities, with the internal exhibition of stimulants, of which camphor and sulphuric ether occupied a prominent place.

It has been not unaptly remarked of Asiatic Cholera, that "it is a disease which begins where all other diseases end—in death;" and numberless cases are to be met with, in which the shock to the nervous system is of such a powerfully depressing nature, as to place the absorbent system at once beyond the pale of any influence. Such cases could not be benefitted by any treatment, however judiciously or assiduously applied; but if, on the contrary, the vitality of the system is not at once paralysed, i the absorbents can act, no matter to how trifling soever an extent, the fairest prospect is afforded to us, through calomel, of rescuing the patient from an otherwise imminent death. The calomel treatment has then something more than a mere claim on us, on the grounds of theory; its practical employment has proved as signally successful in the hands of other medical gentlemen in this city, as it has done in mine.

Montreal, July 30, 1849.—*British American Journal.*

